# A New Proxy Measurement Algorithm with Application to the Estimation of Vertical Ground Reaction Forces Using Wearable Sensors

**DOI:** 10.3390/s17102181

**Published:** 2017-09-22

**Authors:** Yuzhu Guo, Fabio Storm, Yifan Zhao, Stephen A. Billings, Aleksandar Pavic, Claudia Mazzà, Ling-Zhong Guo

**Affiliations:** 1 Department of Automatic Control and Systems Engineering, The University of Sheffield, Mappin Street, Sheffield S1 3JD, UK; yuzhu.guo@sheffield.ac.uk (Y.G.); s.billings@sheffield.ac.uk (S.A.B.); 2 INSIGNEO Institute for in Silico Medicine, The University of Sheffield, Mappin Street, Sheffield S1 3JD, UK; f.storm@sheffield.ac.uk (F.S.);a.pavic@exeter.ac.uk (A.P.); 3 School of Automation Science and Electrical Engineering, Beihang University, Beijing 100191, China; 4 Department of Mechanical Engineering, The University of Sheffield, Mappin Street, Sheffield S1 3JD, UK; 5 The EPSRC Centre for Innovative Manufacturing in Through-life Engineering Services, Cranfield University, Cranfield MK43 0AL, UK; yifan.zhao@cranfield.ac.uk; 6 Vibration Engineering Section, College of Engineering, Mathematics and Physical Sciences, University of Exeter, North Park Road, Exeter EX4 4QF, UK

**Keywords:** proxy measurement, vertical ground reaction force, NARMAX, orthogonal forward regression

## Abstract

Measurement of the ground reaction forces (GRF) during walking is typically limited to laboratory settings, and only short observations using wearable pressure insoles have been reported so far. In this study, a new proxy measurement method is proposed to estimate the vertical component of the GRF (vGRF) from wearable accelerometer signals. The accelerations are used as the proxy variable. An orthogonal forward regression algorithm (OFR) is employed to identify the dynamic relationships between the proxy variables and the measured vGRF using pressure-sensing insoles. The obtained model, which represents the connection between the proxy variable and the vGRF, is then used to predict the latter. The results have been validated using pressure insoles data collected from nine healthy individuals under two outdoor walking tasks in non-laboratory settings. The results show that the vGRFs can be reconstructed with high accuracy (with an average prediction error of less than 5.0%) using only one wearable sensor mounted at the waist (L5, fifth lumbar vertebra). Proxy measures with different sensor positions are also discussed. Results show that the waist acceleration-based proxy measurement is more stable with less inter-task and inter-subject variability than the proxy measures based on forehead level accelerations. The proposed proxy measure provides a promising low-cost method for monitoring ground reaction forces in real-life settings and introduces a novel generic approach for replacing the direct determination of difficult to measure variables in many applications.

## 1. Introduction

The analysis of ground reaction force (GRF) (i.e., the force of interaction between the body, usually the foot, and the ground) is central in many scientific and engineering fields, including biomechanics, medical science, sports science, and robotics [1,2,3,4]. In human biomechanics and humanoid robotics, for example, postural control is critical for understanding balance and locomotion, where the control strategies for bipedal systems heavily rely on the knowledge of the GRF and its point of application, i.e., the centre of pressure (COP). In healthcare, estimating the GRF and joint moments of patients in daily life activities could have substantial clinical impact by providing assessments of pathological gait, fall detection in the elderly, and biofeedback data for home interventions [5,6].

In human biomechanics, standard measuring techniques for GRFs are restricted to laboratory settings, where GRFs can be accurately measured using calibrated force platform systems, but this limits the applicability of the relevant results, which are obtained for one step only. Whereas instrumented treadmills with embedded force platforms allow for accurate multi-step GRF measures, they are still limited to the laboratory setting. Furthermore, some clinical gait features are often triggered by free-living environmental challenges and cannot be replicated in a controlled laboratory environment. Continuous monitoring in unsupervised habitual environments is essentially useful for enhancing diagnostics, monitoring disease progression, measuring the efficacy of intervention, and predicting the risk of falls and cognitive decline [7].

Portable and wearable sensor systems have been developed to allow for the measurement/estimation of the GRFs or foot pressure distributions in a real environment outside a laboratory or in daily life [8,9,10,11,12,13,14,15,16,17]. The output of these systems provides the GRF and its point of application (centre of pressure (COP)) for a variety of applications. However, systems such as force sensitive resistors are still relatively expensive, quite cumbersome to wear and prone to mechanical damage, with the result of a limited applicability outside the specialised research field. Inertial measurement units (IMU) are sensors suitable for long-term monitoring of gait information [18], which would allow overcoming these limitations. It will be of significant importance if the GRF information can be reconstructed from the IMU data.

The problem of the estimation of GRFs without using force plates has been tackled by other authors [12,19,20,21], some of whom yielded results using IMU recordings [22,23,24,25]. However, the applications of these approaches have certain constraints, since most of them require modelling of biomechanical systems to a certain extent so that some data of the body segments (such as limbs) of the particular subject is required [19,20] such as masses, dimensions, and centres of masses. These are therefore heavily subject-dependent and require extensive knowledge for correct modelling. In some cases, such approaches also require data from many IMU sensors; for example, 16 sensors were used for data collection in one such study [25], which limits the applicability within a real-life context. One inertial sensor has been used to estimate some characteristics of GRF, such as the peak values and mean value of the GRF, rather than a full profile of the GRF in a gait cycle [22,23,24]. In these studies, the accelerations were directly used as indicators of the GRF. However, the dynamics between the accelerations and GRF has not been explored.

To this end, a novel and generic proxy measurement method is proposed without regard to the biomechanical modelling of movement. The NARMAX (nonlinear auto-regressive moving average models with exogenous inputs) method [26,27,28,29] is adopted to identify the dynamic relationships between the proxy variables, i.e., the acceleration from IMUs, and the measured vertical GRFs (vGRFs). The NARMAX methods provide linear and/or nonlinear dynamic relationships and models between user-defined inputs and outputs, both pertaining to our problem.

The aim of this study is to introduce a new generic algorithm to provide a proxy measure for unobservable variables. In this specific application, wearable IMU sensors will be used to measure the accelerations at different body levels. The acceleration signals are used as the proxy variables, and the dynamical relationship between vGRF and the accelerations is explored. The new algorithm is then used to estimate vGRF from these accelerations. Based on the new proxy measurements, the predicted vGRFs from the developed dynamic models are compared and evaluated with simultaneously vGRF data obtained from pressure insoles. The proxy measurement of vGRFs is studied for both outdoor specified (controlled straight) walking and outdoor free walking.

## 2. Materials and Methods

Nine healthy volunteers (3 females, 6 males, age 28 ± 3 years old) were recruited for the study. Ethical approval was obtained from the University of Sheffield’s Research Ethics Committee, and the research was conducted according to the declaration of Helsinki. All participants provided written informed consent.

Each participant was asked to wear three IMUs (Opal™, APDM; weight 22 g, size 48.5 mm × 36.5 mm × 13.5 mm) containing a 3-axis accelerometer, a 3-axis gyroscope, and a 3-axis magnetometer. One IMU was positioned on the lower trunk on the fifth lumbar vertebra (L5) with its sensing axes *X*, *Y*, and *Z* pointing downward, to the left and forward, respectively. The other two IMUs were positioned at the seventh cervical vertebra (C7) and forehead (FH), with *X*, *Y*, and *Z* pointing downward, to the right and backward, respectively. The devices measured accelerations at a sampling frequency of 128 Hz, and the accelerometer range was set at ±6 g. It is worth emphasising that only the 3-axis accelerations were used in the study although the sensors can also provide angular velocity and orientation information. Hence, the proxy measures are actually free from the limitations related to gyroscope drifts and magnetic disturbances.

Two pressure-sensing insoles (F-Scan 3000E, Tekscan^TM^, South Boston, MA, USA) were used to obtain the vGRF reference. The insoles were cut to fit tightly into each participant’s shoe. They were calibrated using a step calibration technique according to the manufacturer’s instructions. The sampling frequency was set at 128 Hz. A Fourier analysis of the vGRF time series showed that all main frequency components had a frequency lower than 10 Hz. Therefore, a sample frequency greater than 64 Hz was deemed high enough to characterise the main frequency spectral.

Subjects completed two walking tasks in the conditions detailed in Table 1. The IMU and pressure insoles data were collected during each task. For the outdoor free walking task, participants were instructed to walk freely in the city centre without any restrictions regarding route or walking speed, and avoiding stairs. For the outdoor controlled walking, participants were asked to walk back and forth along a 50 m walkway at their preferred speed. More details about the protocol are available in [30]. The outdoor free walking conditions had the potential of recording the participant’s turns in addition to straight line walking, both of which were included in the analysis. Data recorded during resting or transitory periods were excluded from the analysis. A vertical jump was used as a synchronising event between the IMUs and the insoles in order to realign the signals coming from the two instruments at the beginning of each trial. The equivalency of the nominal sampling frequency of the two instruments was verified, and the mismatch was corrected for the 15 min outdoor free walking (OFW) data by realigning the signals every 2 min. This procedure was not needed in the outdoor controlled walking (OCW) tasks, which lasted less than 2 min.

### 2.1. The General Idea of the Proxy Measure

Once the accelerations and vGRF signals have been recorded, the relationship between the accelerometer signals and the insole measured vGRF can be modelled. The higher-order cross-correlation nonlinear detection method [27,31] was applied and indicated that a linear model is not sufficient to describe the relationships between these two types of signals. Therefore, a nonlinear dynamic model was developed in this study.

The accelerations are defined on the sensor frame, while the vGRFs are defined in a ground frame. The coordinate transformation can be represented by a linear map:

(1) $${\vec{a}}_{global}=T\left(\vartheta \right){\vec{a}}_{sensor}$$

where the orientation ϑ of the sensor frame is known. 

The relationship between the vGRF and the accelerations can be described in the global frame by a function given as

(2) $$vGRF=f_{0}\left({\vec{a}}_{global}\right)=f_{0}\left(T\left(\vartheta \right){\vec{a}}_{sensor}\right)$$

However, the transform matrix Τ(ϑ) may change over time, due to changes in the orientation of the sensor. It is then possible to define a relationship based on the orientation and accelerations as

(3) $$vGRF=f_{1}\left(\vartheta ,{\vec{a}}_{sensor},g\right)$$

where g is a constant representing gravity. The effect of gravity can be considered as an implicit parameter in the model, as detailed in the discussion.

A further assumption is that the entries in the coordinate transformation matrix can be expressed as functions of the time-varying accelerations ${\vec{a}}_{sensor}\left(t\right)$ and of the associated time delays ${\vec{a}}_{sensor}\left(t-\tau_{i}\right),\;\tau_{i}>0$. 

Equation (3) can then be rewritten as a function of the accelerations:

(4) $$vGRF\left(t\right)=f\left({\vec{a}}_{sensor}\left(t\right),{\vec{a}}_{sensor}\left(t-\tau_{1}\right),\cdots ,{\vec{a}}_{sensor}\left(t-\tau_{L}\right)\right)$$

For the time series, the discrete time relationship reads as

(5) $$vGRF\left(k\right)=f\left({\vec{a}}_{sensor}\left(k\right),\;{\vec{a}}_{sensor}\left(k-1\right),\cdots ,{\vec{a}}_{sensor}\left(k-L\right)\right)$$

where *L* represents the maximum time delay and *k* denotes the *k*th sample instance.

The function form $f\left({\vec{a}}_{sensor}\right)$ is usually unknown. However, according to the Stone–Weierstrass-like approximation theorems [32], the function can often be approximated as the linear superposition of a set of known basis functions $\phi_{i}\left({\vec{a}}_{sensor}\right)$ as

(6) $$vGRF=\sum_{i}^{n}\theta_{i}\phi_{i}\left({\vec{a}}_{sensor}\left(k\right),\;{\vec{a}}_{sensor}\left(k-1\right),\cdots ,{\vec{a}}_{sensor}\left(k-L\right)\right)$$

The model structure and the associated parameters $\theta_{i}$ can be learned using the orthogonal forward regression algorithm.

Based on the model structure, the accelerations measured in the sensor reference frame can be directly used without any frame transformation. The underlying nonlinear relationship, in fact, will be learned by the iterative orthogonal forward regression algorithm (iOFR) algorithm. Thereafter, the vGRF can be predicted based on the obtained model using only the output of an accelerometer outputs.

The results in Section 3 validate the assumptions in our method and show that this kind of model structure is capable of describing the underlying nonlinear dynamic connections between the acceleration and vGRF even in an outdoor free-style walking scenario.

### 2.2. Decomposition of the Sensor Signals

Since we wanted to use the acceleration readings a from one single wearable sensor as a proxy measurement for vGRFs of both feet, a key step in this study was to separate the single input variable a into two components: one reflects the walking when the left leg has dominant pressure on the ground ($a_{left}$), and the other reflects the walking when the right leg has dominant pressure on the ground. The two components were defined by introducing two membership functions as follows.

(7) $$\begin{array}{cc} w_{left}=\frac{GRF_{left}}{GRF_{left}+GRF_{right}}, & w_{right}=\frac{GRF_{right}}{GRF_{left}+GRF_{right}} \end{array}$$

**Figure 1:**
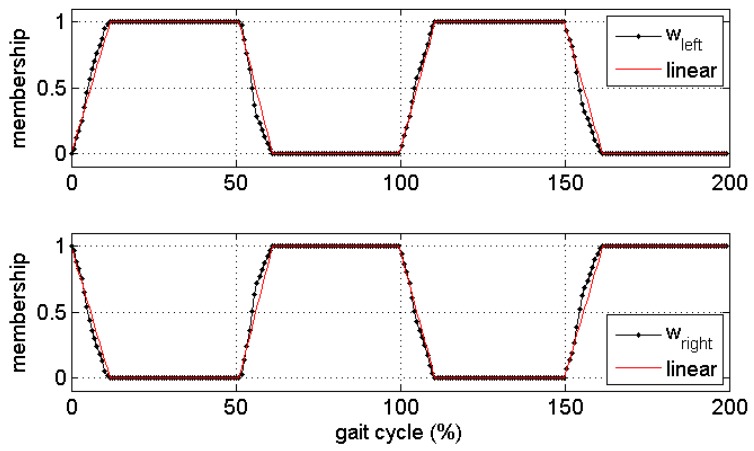
Approximated membership functions of the vertical ground reaction force (vGRF). The true memberships calculated with Equation (7) are shown in black dotted lines; the approximations of the membership function with gait events are shown in red lines. The upper panel shows the left membership function, which corresponds to left single support phases; the lower panel shows the right membership function corresponding to the right single support phases.

The left and right components can then be split by the defined membership functions as

(8) $$\begin{array}{cc} a_{left}=a\cdot w_{left}, & a_{right}=a\cdot w_{right} \end{array}$$

where a is the acceleration recordings and the “·” represents the point-wise multiplication operation. An approximation to the membership functions can be estimated using gait events such as the IC (initial foot contact) and FC (final foot contact) instants calculated from the insole pressure sensor information. The membership is set as 1 in the single support phase and 0 in the swing phase. These two values are linearly connected in the double support phases. The left and right membership functions can then be approximately obtained, and the acceleration recordings can be decomposed into the left and right components. Figure 1 shows the calculated and approximated membership functions for the IMU signal decomposition in two gait cycles. The gait events can also be obtained from an extra inertial sensor at the pelvis or shank level [30,33]. This will release the limitation in the applications. In this study, the gait events were detected using the ground reaction force with a 10 N threshold [34] to avoid errors possibly introduced in calculating the gait events form the IMU.

### 2.3. The Proxy Model Development

Once the left and right components of the acceleration signals were obtained, a special type of NARMAX model [27], based on expansions of the input only, giving essentially a Volterra series expansion or a nonlinear moving average (NMA) model [35], was used for the derivation of the vGRF model. This expansion provides a general representation of nonlinear dynamics, where only the nonlinearities in the input variables are involved. This simplifies the prediction of the vGRF from only the current and past acceleration recordings.

A discrete NMA or Volterra series model can be defined as

(9) $$\begin{array}{l} y\left(k\right)=\sum_{n=0}^{N}y_{n}\left(k\right)\;,\\ y_{n}\left(k\right)=h_{n}\left(m_{1},m_{2},\cdots ,m_{n}\right)\prod_{i=1}^{n}u\left(k-m_{i}\right) \end{array}$$

where u(k) and y(k) are the system input and output, respectively, and $m_{i}$ represents time delays. The *n*th kernel $h_{n}\left(m_{1},m_{2},\cdots ,m_{n}\right)$ characterises the weight of the *n*th nonlinearity in the system response. The discrete Volterra series model can be rewritten in the general NARMAX form as follows:

(10) $$y\left(k\right)=F\left[u\left(k-1\right),u\left(k-2\right),\cdots ,u\left(k-n_{u}\right)\right]$$

where F is a multivariate polynomial function and *n_u_* denotes the maximum time delay of the input.

In the context of GRF prediction, the left and right components of three measured perpendicular accelerations $\left\{a_{x},a_{y},a_{z}\right\}$ defined in the IMU frame are the inputs, and the vGRFs $\left\{GRF_{left},GRF_{right}\right\}$ from left or right foot are the output. That is, a total number of six inputs $\left\{a_{x,left},a_{x,right},a_{y,left},a_{y,right},a_{z,left},a_{z,right}\right\}$ was included in the model.

Once the maximum time delay is specified, the model structure can be constructed based on Equation (3). However, the model can include a huge number of terms; for example, the number of terms in the model is 5778 when the maximum time lag is 18 samples. This may lead to over-fitting of the data or numerical ill-conditioning in parameter estimation.

The OFR algorithm and the associated variants have been proven able to efficiently determine a sparse model structure and have been widely used in a wide range of applications [27]. Here, an improved OFR algorithm, an iterative OFR, was used to identify the model structure and explore the relationship between the desired vGRF and the proxy measurements [28,29]. A more detailed discussion of the iterative OFR algorithms can be found in [28].

Once a reliable model is built, the vGRF can be reconstructed with the chosen wearable sensor information only. The final model structure in this study included only the nonlinear moving average part of the proxy measurements and no information about the output, i.e., the vGRF was used. This made the prediction of the GRF much easier, and the prediction error would not accumulate in the predicted GRF.

The same procedure was applied to the 9 participants and two walking tasks, respectively, to build subject-specific proxy models. The subject-specific models produced more accurate estimation of vGRF than an average model, which was built by pooling all subject data. The subject-specific models performed better because subject- and task-specific information was characterised by the models.

### 2.4. Accuracy Analysis

To assess the performance of the models, the predicted GRFs were compared with the pressure insole recordings. Following the definitions given in [36], the differences were quantified using the root mean squared error (RMSE):

(11) $$RMSE=\sqrt{\frac{1}{N}\sum_{k=1}^{N}(y(k)-\hat{y}(k){)}^{2}}$$

where y(k) and $\hat{y}(k)$ are the predicted and measured vertical GRFs, respectively, and N is the sampling number for comparison. The relative RMSE (rRMSE) with respect to the average peak-to-peak amplitude between two values was also used to quantify the performance of the prediction:

(12) $$rRMSE=\frac{RMSE}{\left(\max\left(y\left(k\right)\right)-\min\left(y\left(k\right)\right)+\max\left(\hat{y}\left(k\right)\right)-\min\left(\hat{y}\left(k\right)\right)\right)/2}$$

The ranges for the maximum and minimum were calculated over the number of samples used for validation.

The predicted vGRFs were compared with the insole measured reference signals for each gait cycle. The mean and standard deviations of the prediction errors over the gait cycles were compared for each individual and each task. A Student’s *t*-test was adopted to analyse the effects of different walking tasks, sensor locations, and inter-subject variability on the accuracy of the proxy measure.

## 3. Results

The data from OCW and OFW were split into a training set and a test set. Half of the data were used to identify the model, and the remaining half were used to validate the model and analyse the prediction errors. The total OCW data included 23,040 samples, which were about 172 gait cycles after removing the resting or transitory periods. The total OFW data included 92,160 samples, about 688 gait cycles.

It is worth emphasising that all of the prediction error analysis given below was based on the test set only, excluding the data used for training the models. The results illustrated the predictive ability of the obtained proxy model. A large training set was used to include as rich as possible gait variability for the purpose of improving the model predictive performance under different cases.

### 3.1. Proxy Measurement of vGRF Based on the Waist Level Sensor Signal

The six split acceleration signals $\left\{a_{x,left},a_{x,right},a_{y,left},a_{y,right},a_{z,left},a_{z,right}\right\}$ were used to fit the left and right vGRF by the NARMAX Model (10). The iterative OFR algorithm was used to detect the model structure and estimate the associated parameters. Half of the data were used to identify the model, and the other half were used to validate the predictive power of the obtained model.

A cross-correlation analysis between waist acceleration and total GRF indicated that most of the time delays between the acceleration and the total vGRF were fewer than 18 samples. Hence, a maximum time lag of 18 was used to build the NARMAX model. All of the left and right components of the waist level accelerations with a time lag of less than 18 were used to construct a term dictionary consisting of all of the combinations of $\left\{a_{x,left}\left(k\right),\cdots ,a_{x,left}\left(k-5\right),a_{x,right}\left(k\right),\cdots ,a_{x,right}\left(k-18\right),\cdots ,a_{z,right}\left(k-18\right)\right\}$, up to second-order polynomial terms, that is $a_{i}^{p}\left(k-n_{i}\right)a_{j}^{q}\left(k-n_{j}\right)$, 0≤p+q≤2, $0\leq n_{i},n_{j}\leq 18$, where $a_{i},a_{j}\in \left\{a_{x,left},a_{x,right},a_{y,left},a_{y,right},a_{z,left},a_{z,right}\right\}$. A 64-term NARMAX model was obtained for both left and right vGRFs.

**Figure 2:**
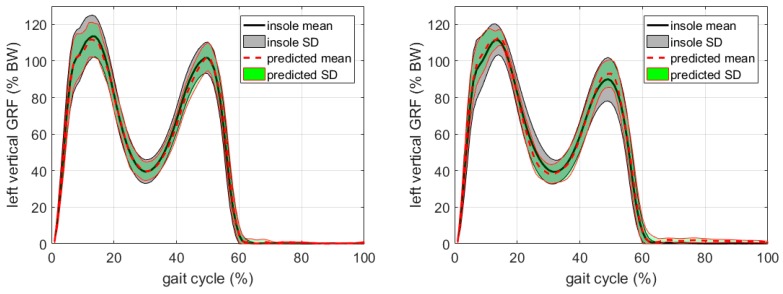
Proxy measured GRF (mean red thick broken line ± 1 SD red thin lines) compared with insole data (mean (black thick line) ± 1 SD (grey shaded area)); left figure: outdoor controlled walking (OCW), right figure: outdoor free walking (OFW).

A typical proxy model prediction of the vGRFs for the OCW and OFW tasks is shown in Figure 2, based on the data from Participant No. 1. The proxy measures are significantly correlated with the insole measures, with cross-correlation coefficients ρ=0.993 (*p* < 0.01) for OCW and ρ=0.990 (*p* < 0.01) for OFW. Similar results were obtained using the data from other participants.

**Figure 3:**
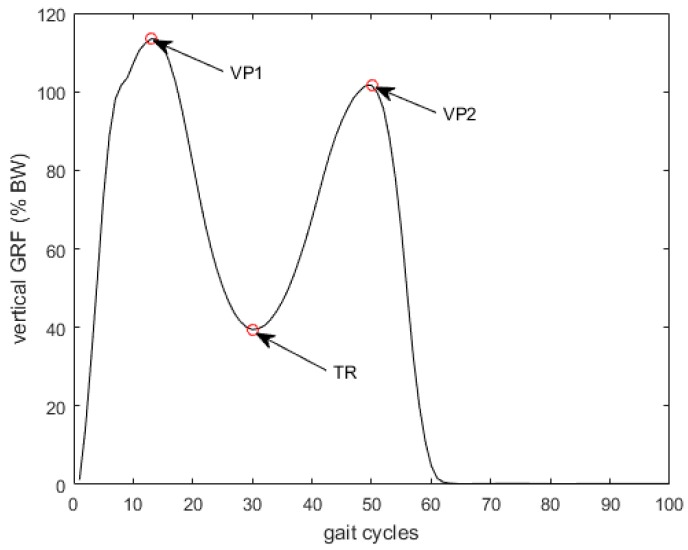
Illustration of the ground reaction force vertical peaks (VP1 and VP2) and trough (TR).

More detailed prediction errors for the OCW and OFW tasks are shown in Table 2 and Table 3. The prediction errors for full gait cycles, single support phases, double support phases, and three critical points (two vertical peaks, VP1, VP2, and one trough values, TR in Figure 3) are listed. The mean relative prediction errors (in rRMSE %) were less than 5.2% for the OCW. Generally, the prediction errors for OFW were less than 7.0%, which is greater (with *p* = 0.01) than OCW. This may be because both the walking direction and speed were restricted in the OCW, and the consistency between the training and the test data was better than the OFW data. Therefore, the predictions of the test data in OCW were more accurate than those in the OFW cases. The average prediction errors over all participants were 3.8% and 5.0% for OCW and OFW, respectively. The highest prediction error happened at VP2 for both OCW and OFW cases. The average prediction errors were 4.6% and 6.2%, respectively, which were larger than the overall prediction error (with *p* = 0.16 and 0.14, respectively). This means the model prediction at VP2 was less accurate than the overall performance.

### 3.2. The Effect of Sensor Location

**Figure 4:**
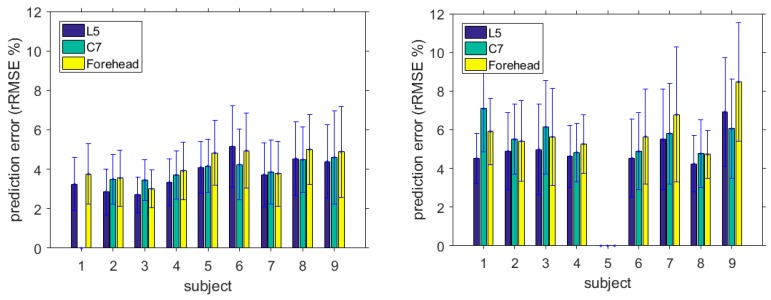
Effects of sensor location on prediction errors; left figure: OCW, right figure: OFW.

The other two accelerations measured at the cervical (C7) and forehead (FH) levels were also used as the proxy variable to estimate vGRFs. The results are shown in Table 4, Table 5, Table 6 and Table 7. The overall mean of the prediction error for OCW based on C7 and FH accelerations was 4.0% and 4.2%, respectively, which was greater (with *p* = 0.67 and *p* = 0.29, respectively) than the prediction error 3.8% produced by the L5 sensor. This could be because the movement of the waist in the OCW was more stable. Similar results can be observed in the OFW case. The overall mean of the prediction error for OFW-based C7 and FH was 5.6% and 6.0%, respectively, which were greater (with *p* = 0.17 and 0.08) than the prediction error produced by the L5 sensor. In sum, the L5 proxy measure had the best performance compared to the other two. The difference among the performance of different sensors was not significant with *p* > 0.29 in the OCW cases, while there were relatively larger difference in the performances for the OFW tasks. The comparison of the full gait cycle prediction errors based on different sensor locations is summarised in Figure 4.

### 3.3. Inter-Subject Variability

The inter-subject variances for OCW were small for all three models (0.68, 0.19, and 0.55). In the OFW cases, the inter-subject variances were relatively small (0.73 and 0.65) using the L5 and C7 models. The OFW variance based on FH model was 1.36, which was greater than those of the other two sensor positions. Hence, the C7 model kept a low inter-subject variance for both OCW and OFW, and the performance was more stable than the other two proxy models. This can also be inferred from the results shown in Figure 4.

In summary, the L5 sensor-based proxy model showed the minimum model prediction errors and the C7 model the smallest inter-subject variability. There were no significant differences in the performances of the proxy models based on three different sensor locations.

## 4. Discussion

When analysing an individual’s gait, the knowledge of GRFs is very important as input for the joint mechanics [37]. The gold standard method of measuring GRFs is based on the use of a force plate. The instrumented treadmills can overcome the restrictions on the number of consecutive gait cycles that can be analysed. The studies to predict GRFs using motion data or kinematic data of the subjects have been another focus of the research [5,11,12,19,20,36,38,39,40,41]. However, most of these methods are restricted to gait laboratory settings. In this paper, we have demonstrated a low-cost proxy measurement method to accurately predict the vertical GRFs using only one inertial sensor.

This study aimed at reconstructing the vGRF under each foot using as few sensor recordings as possible, preferably from one wearable sensor. In this way, we could achieve a good prediction of vGRF with extremely low cost. To this end, the accelerations recorded at three different levels were investigated. The three locations were forehead, base of neck and lumbar. It was shown that the L5 model has smaller prediction error and relatively less inter-subject variability. Another advantage of using the L5 sensor is that the gait events, which were used in splitting the vGRF signals, can be detected from the waist level IMU information based on the method in [30], and no extra sensor information is needed.

The quality (prediction accuracy) of the proxy measures of the vGRF is comparable to the direct measurement of vGRF [11] and measurement based on the inverse dynamics method [36]. In [11], the ground reaction kinetics were estimated, including three ground reaction forces, two centres of pressure, and vertical torque. The average normalised prediction error (rRMSE) for the vGRF in the intra-day single-task is less than 3.5% and in the multi-task prediction error less than 4.2%. In [36], the relative RMSE prediction error for vGRF was about 6.0%. Both of the above studies were conducted in an indoor condition and with fixed walking speed. Our average prediction error for fixed walking speed tasks is about 3.8%, and the free waking without speed restriction is about 5.0%. Furthermore, our study was in an outdoor condition, which is more challenging.

In the proposed method, only the accelerations from the inertial sensor were used to build the model. Other sensor information, for example, the angular velocities, has also been tested in the study. Results showed that including the angular velocities is of little help in improving prediction accuracy. On the contrary, using less information will reduce the complicity of the model and increase the model’s robustness. A preliminary study showed that the angular velocities played an important role in the prediction of COP.

The technique used in this study is decomposing acceleration signals into left and right components for the purpose of predicting both left and right vGRFs at the same time. This procedure further enhanced the correlations between the model predictions and vGRFs. This is critical for the prediction performance of the proposed approach. The decomposition conducted in this paper was based on the gait event information, e.g., heel-strike and toe-off. We chose to extract this information from the pressure insoles, being more accurate for this purpose, to isolate a possible source of additional error from the final estimate of the model outputs. This can be a limitation of the proposed method because this information may not be readily available. However, this information can be obtained from inertial sensors located on the pelvis or ankles in real applications [30,33]. For example, the inertial sensor signals at the L5 level can be used for both splitting the data and building the proxy model.

We have shown how the NARMAX modelling approach can be used to identify a simple, but nonlinear proxy model for predicting vGRFs of both feet, during normal daily outdoor walking. The task investigated in this paper could have been achieved using other machine learning approaches, such as supervised artificial neural networks (ANNs). For instance, ANNs have been used to predict the joint load in motion [42,43] and the ground reaction forces during gait [19]. However, these approaches tend to be slow in learning, especially when using large input spaces and, more importantly, generate opaque models that are difficult to visualise and analyse. In contrast, the NARMAX modelling methodology produces transparent mathematical functions that are directly related to the task.

The model needs to be validated for the prediction of ground reaction forces during more daily living activities. The application of the developed method in predicting mediolateral and anterior–posterior ground forces is of interest. It is noteworthy to recall that this study involved only young healthy volunteers, whereas upper body movements and stability tends to change with aging and pathologies [44]. Therefore, further investigations are needed to translate the results of this study to other populations, for example, other age groups or groups with pathological gaits. However, the method is expected to be applicable to other groups because of the subject-specified modeling procedure.

A proxy measurement method is used in this study where the vGRFs are indirectly estimated through measuring the proxy variables, namely the accelerations, which are much easier to obtain in out-of-laboratory settings. The most common use of proxy measurement is that of substituting a measurement of one variable that is inexpensive and/or easily obtainable for a different variable that would be more difficult or costly, if not possible, to collect. Proxy measurements have been widely used in the social sciences, but rarely in engineering applications. Therefore, the methodology and results in this study could have important implications beyond ground reaction force prediction with many applications in medicine. A similar proxy measurement strategy can be implemented in other engineering applications that involve unobservable and/or expensively measurable states and variables.

## 5. Conclusions

In this study, a proxy measurement method has been proposed to estimate the vGRFs in non-laboratory settings. Inertial sensor information has been used as proxy variables, and the nonlinear dynamic relationship between the vGRF and accelerations has been revealed using a NARMAX model. The proposed method is easy to proceed and provides a low-cost but reliable proxy measure of vGRFs in non-laboratory settings. This makes the long-term monitoring of the gait characteristics in a free-living condition possible. Another advantage of the new methods is that it provides an explicit model for the dynamic relationships between the accelerations at different body levels and the vGRFs. This can be used for further model-based analyses, for example, the nonlinear spectral analysis, to explore some new gait characteristics which cannot be obtained using a simple statistical method [27,45].

In future research, the obtained models will be used for predicting ground reaction forces during various activities of daily living. The application of the developed method in predicting mediolateral and anterior–posterior ground forces is of interest. Further studies will involve other age groups or disease-related ground reaction force predictions such as Parkinson’s disease. While the present study focuses on using the new proxy algorithm in the application of GRFs, the ideas are applicable over a very wide spectrum of problems and can be used for generic proxy measurement reconstructions of other immeasurable signals.

## Figures and Tables

**Table 1:** Summary of the walking conditions performed during the experiments, with the acronym, description, and duration or repetition.

Condition	Acronym	Description	Duration/Repetitions
Outdoor controlled walking	OCW	Walking at preferred speed along a 50.0 m-long walkway	Six repetitions
Outdoor free walking	OFW	Walking along footpaths open to the public in the city centre without any restrictions in route or walking speed, avoiding stairs	Fifteen minutes

**Table 2:** Mean and SD of the prediction errors for OCW (rRMSE %) based on fifth lumbar vertebra (L5) acceleration.

Participant	Full Gait Cycle	Single Support	Double Support	VP1	VP2	TR
No. 1	3.2 ± 1.3	4.2 ± 1.7	3.4 ± 1.5	5.1 ± 4.0	2.9 ± 3.2	3.5 ± 3.4
No. 2	2.9 ± 1.2	3.6 ± 1.5	3.0 ± 1.2	4.0 ± 3.4	3.0 ± 2.8	3.6 ± 2.4
No. 3	2.7 ± 0.9	3.5 ± 1.2	3.9 ± 1.6	3.4 ± 2.5	3.8 ± 2.8	2.7 ± 2.1
No. 4	3.3 ± 1.2	4.2 ± 1.5	3.7 ± 1.5	4.0 ± 3.0	4.1 ± 3.2	3.7 ± 3.0
No. 5	4.1 ± 1.3	5.3 ± 1.7	4.0 ± 1.4	3.8 ± 3.2	6.2 ± 5.2	4.6 ± 2.7
No. 6	5.1 ± 2.1	6.4 ± 2.6	5.9 ± 2.2	6.1 ± 4.6	7.0 ± 5.1	5.3 ± 5.1
No. 7	3.7 ± 1.6	4.8 ± 2.1	5.1 ± 2.5	4.3 ± 2.8	4.2 ± 3.0	4.4 ± 3.6
No. 8	4.5 ± 1.9	5.9 ± 2.5	4.2 ± 1.4	3.8 ± 3.8	6.3 ± 4.9	5.7 ± 4.3
No. 9	4.4 ± 1.9	5.6 ± 2.4	4.2 ± 1.7	4.2 ± 3.3	4.0 ± 2.9	5.6 ± 3.7

**Table 3:** Mean and SD of prediction errors for OFW (rRMSE %) based on L5 acceleration.

Participant	Full Gait Cycle	Single Support	Double Support	VP1	VP2	TR
No. 1	4.5 ± 1.3	5.8 ± 1.7	5.2 ± 1.9	4.7 ± 3.9	6.5 ± 4.2	5.6 ± 4.1
No. 2	4.9 ± 2.0	6.1 ± 2.5	5.1 ± 1.9	5.9 ± 4.7	6.0 ± 4.6	5.7 ± 3.8
No. 3	5.0 ± 2.3	6.4 ± 3.0	6.0 ± 2.9	6.0 ± 4.7	5.4 ± 4.1	5.6 ± 5.5
No. 4	4.6 ± 1.6	5.9 ± 2.1	6.0 ± 2.4	4.7 ± 4.3	4.5 ± 3.5	6.7 ± 5.3
No. 5	The insole sensor produced incorrect data in this task					
No. 6	4.5 ± 2.0	5.8 ± 2.6	4.7 ± 2.3	3.8 ± 3.7	4.7 ± 3.9	5.8 ± 4.4
No. 7	5.5 ± 2.6	7.2 ± 3.4	7.3 ± 3.5	5.0 ± 4.0	6.9 ± 5.8	6.9 ± 5.6
No. 8	4.2 ± 1.5	5.5 ± 1.9	3.9 ± 1.5	5.4 ± 4.1	5.1 ± 3.7	3.2 ± 2.5
No. 9	6.9 ± 2.8	8.8 ± 3.6	7.8 ± 3.5	11.0 ± 9.5	10.3 ± 7.8	6.7 ± 5.1

**Table 4:** Mean and SD of prediction errors for OCW (rRMSE %) based on seventh cervical vertebra (C7) acceleration.

Participant	Full Gait Cycle	Single Support	Double Support	VP1	VP2	TR
No. 1	Corrupted data from the IMU sensors					
No. 2	3.5 ± 1.3	4.4 ± 1.6	3.8 ± 1.7	4.0 ± 2.8	4.6 ± 3.8	5.0 ± 2.9
No. 3	3.4 ± 1.0	4.4 ± 1.4	4.3 ± 1.5	4.1 ± 3.3	4.4 ± 3.2	4.0 ± 2.9
No. 4	3.7 ± 1.2	4.7 ± 1.6	4.3 ± 1.6	4.5 ± 3.5	3.8 ± 3.2	3.7 ± 2.6
No. 5	4.2 ± 1.4	5.4 ± 1.8	4.3 ± 1.6	4.5 ± 3.8	5.3 ± 4.4	4.7 ± 3.3
No. 6	4.2 ± 1.8	5.3 ± 2.2	4.8 ± 1.9	3.2 ± 2.8	5.4 ± 4.2	5.2 ± 4.8
No. 7	3.9 ± 1.6	5.0 ± 2.2	4.7 ± 1.8	3.5 ± 2.7	5.3 ± 4.1	4.6 ± 3.3
No. 8	4.5 ± 1.7	5.8 ± 2.2	4.7 ± 1.9	3.8 ± 3.4	6.0 ± 4.8	6.0 ± 4.2
No. 9	4.6 ± 2.4	5.9 ± 3.0	4.5 ± 2.7	4.2 ± 3.1	5.5 ± 5.5	4.5 ± 3.7

**Table 5:** Mean and SD of prediction errors for OFW (rRMSE %) based on C7 acceleration.

Participant	Full Gait Cycle	Single Support	Double Support	VP1	VP2	TR
No. 1	7.1 ± 2.2	9.0 ± 2.8	6.8 ± 3.4	7.7 ± 6.4	11.3 ± 7.4	7.4 ± 5.7
No. 2	5.5 ± 1.8	7.0 ± 2.3	6.5 ± 2.7	6.9 ± 5.3	8.5 ± 6.1	5.1 ± 4.1
No. 3	6.1 ± 2.4	7.9 ± 3.1	6.2 ± 2.8	6.0 ± 5.0	9.3 ± 7.7	6.8 ± 5.4
No. 4	4.8 ± 1.5	6.1 ± 1.9	5.3 ± 2.1	4.4 ± 3.8	7.5 ± 5.1	5.8 ± 4.0
No. 5	Corrupted data from the pressure insoles					
No. 6	4.9 ± 2.0	6.3 ± 2.6	6.1 ± 3.0	3.8 ± 3.7	8.4 ± 7.2	4.5 ± 3.8
No. 7	5.8 ± 2.6	7.6 ± 3.4	7.3 ± 3.2	5.2 ± 5.1	9.4 ± 7.4	6.3 ± 5.1
No. 8	4.8 ± 1.7	6.2 ± 2.3	4.9 ± 2.1	7.9 ± 6.1	6.2 ± 4.2	4.1 ± 3.2
No. 9	6.1 ± 2.6	7.6 ± 3.3	7.1 ± 3.5	5.6 ± 5.5	10.3 ± 7.1	6.1 ± 4.3

**Table 6:** Mean and SD of prediction errors for OCW (rRMSE %) based on forehead (FH) acceleration.

Participant	Full Gait Cycle	Single Support	Double Support	VP1	VP2	TR
No. 1	3.8 ± 1.5	4.8 ± 2.0	4.1 ± 1.7	5.1 ± 4.0	4.1 ± 3.7	3.7 ± 2.6
No. 2	3.6 ± 1.4	4.5 ± 1.8	3.9 ± 1.7	4.5 ± 3.7	4.0 ± 2.8	4.5 ± 2.8
No. 3	3.0 ± 1.0	3.9 ± 1.2	4.2 ± 1.4	3.8 ± 2.9	4.3 ± 3.0	3.2 ± 2.2
No. 4	3.9 ± 1.5	4.8 ± 1.9	4.4 ± 1.4	4.9 ± 4.7	3.9 ± 3.1	3.3 ± 2.7
No. 5	4.8 ± 1.6	6.2 ± 2.1	4.9 ± 1.8	4.9 ± 3.6	6.1 ± 4.6	5.9 ± 3.7
No. 6	4.9 ± 1.9	6.2 ± 2.4	5.5 ± 2.5	6.1 ± 5.3	6.8 ± 5.5	4.8 ± 3.4
No. 7	3.8 ± 1.6	4.9 ± 2.2	4.9 ± 2.1	5.1 ± 3.7	5.4 ± 4.4	3.6 ± 2.7
No. 8	5.0 ± 1.8	6.5 ± 2.3	5.2 ± 2.0	4.3 ± 3.6	6.8 ± 5.3	6.3 ± 4.1
No. 9	4.9 ± 2.3	6.1 ± 3.0	4.7 ± 2.0	4.2 ± 2.9	5.6 ± 4.8	6.6 ± 4.4

**Table 7:** Mean and SD of prediction errors for OFW (rRMSE %) based on FH acceleration.

Participant	Full Gait Cycle	Single Support	Double Support	VP1	VP2	TR
No. 1	5.9 ± 1.7	7.5 ± 2.2	7.1 ± 3.0	6.3 ± 5.1	8.0 ± 5.2	4.8 ± 3.9
No. 2	5.4 ± 2.1	6.8 ± 2.6	6.0 ± 2.5	7.2 ± 5.6	6.1 ± 4.6	5.7 ± 3.9
No. 3	5.6 ± 2.5	7.2 ± 3.3	6.5 ± 3.2	7.8 ± 6.0	5.4 ± 4.2	5.9 ± 5.3
No. 4	5.3 ± 1.5	6.6 ± 2.0	6.2 ± 2.0	5.6 ± 4.6	4.3 ± 3.4	8.1 ± 5.1
No. 5	Corrupted data from the pressure insoles					
No. 6	5.6 ± 2.5	7.3 ± 3.2	6.2 ± 3.1	6.3 ± 5.1	4.9 ± 4.2	7.4 ± 5.5
No. 7	6.8 ± 3.5	8.9 ± 4.6	9.5 ± 5.3	6.1 ± 5.0	7.1 ± 5.5	8.1 ± 6.8
No. 8	4.7 ± 1.2	6.1 ± 1.6	5.5 ± 1.9	5.4 ± 4.2	7.0 ± 4.1	3.9 ± 3.1
No. 9	8.5 ± 3.1	9.8 ± 3.4	7.8 ± 2.9	12.7 ± 9.2	8.5 ± 6.4	6.7 ± 5.2

## Supplementary Materials

The data used in this paper are available online at https://sheffield.figshare.com/articles/Data_for_the_paper_A_new_proxy_measurement_algorithm_with_applications_to_estimation_of_vertical_ground_reaction_forces_using_wearable_sensors_/5341309.
